# Supplementary material for: Fabricated AIE-Based Probe to Detect the Resistance to Anoikis of Cancer Cells Detached from Tumor Tissue
Source: Cells. 2022 Nov 3;11(21):3478. doi: 10.3390/cells11213478 (PMC9654787; doi:10.3390/cells11213478)
Supplement: Supplementary file 1 [file cells-11-03478-s001.zip › cells-1902572-SI.pdf]

Supplementary information

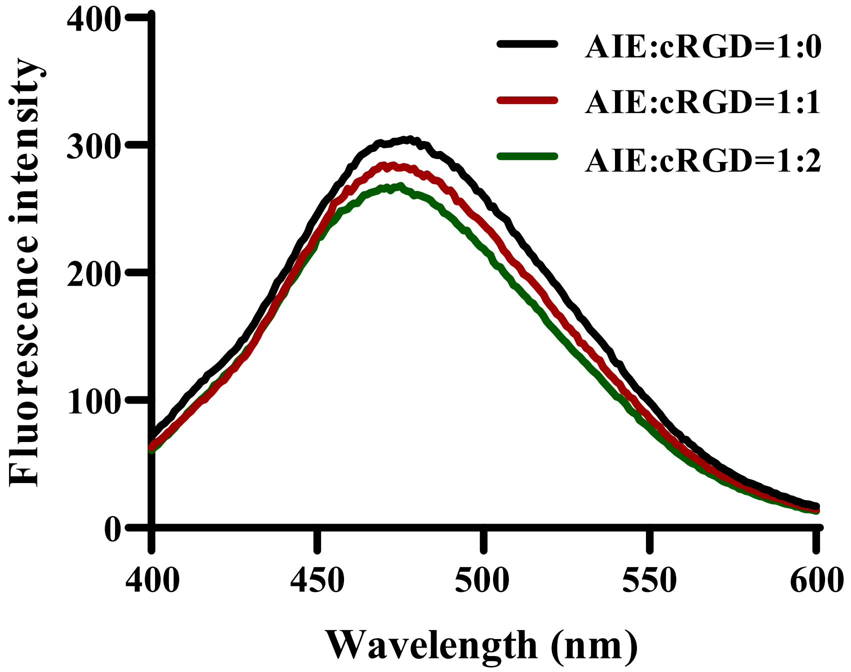

Figure S1. Fluorescence spectra of different proportion of AIEgens with cRGD. The different proportions of AIEgens and cRGD are AIE/cRGD=1/0 (blank line), AIE/cRGD=1/1 (red line), AIE/cRGD=1/2 (green line), respectively.

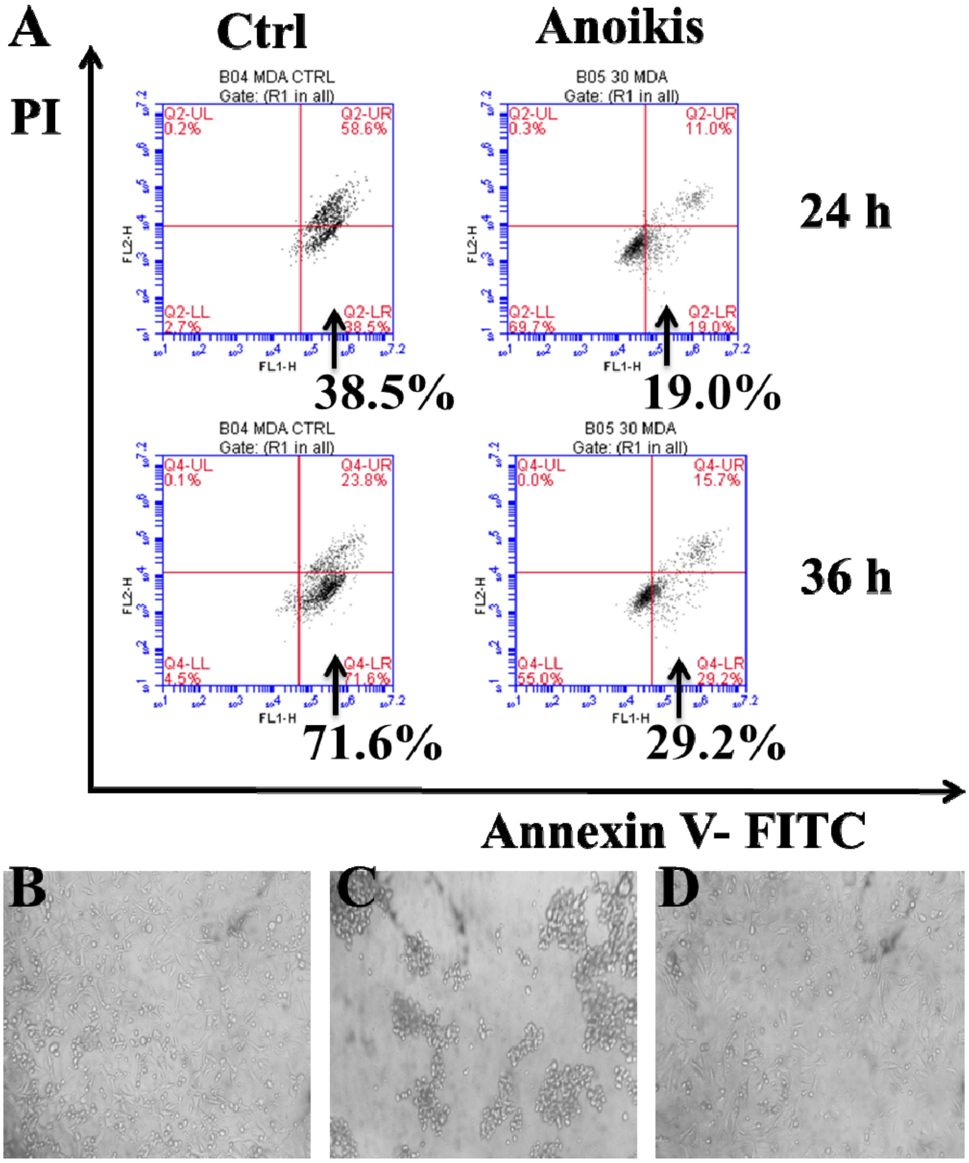

Figure S2. (A) The effects of cell apoptosis on MDA-MB-231 cells cultured for 24 h, 36 h, in six-well cell culture plates treated with poly-HEMA (30 mg/ml). (B-D) The cells morphology change in MDA-MB-231 cells. (B) Cultured for 24 h in untreated six-well cell culture plates. (C) Cultured for 24 h in six-well cell culture plates treated with poly-HEMA (30 mg/ml). (D) Cultured for 24 h in six-well cell culture plates treated with poly-HEMA (30 mg/ml), then transferred some of the cells to untreated six-well cell culture plates incubation for 24 h.

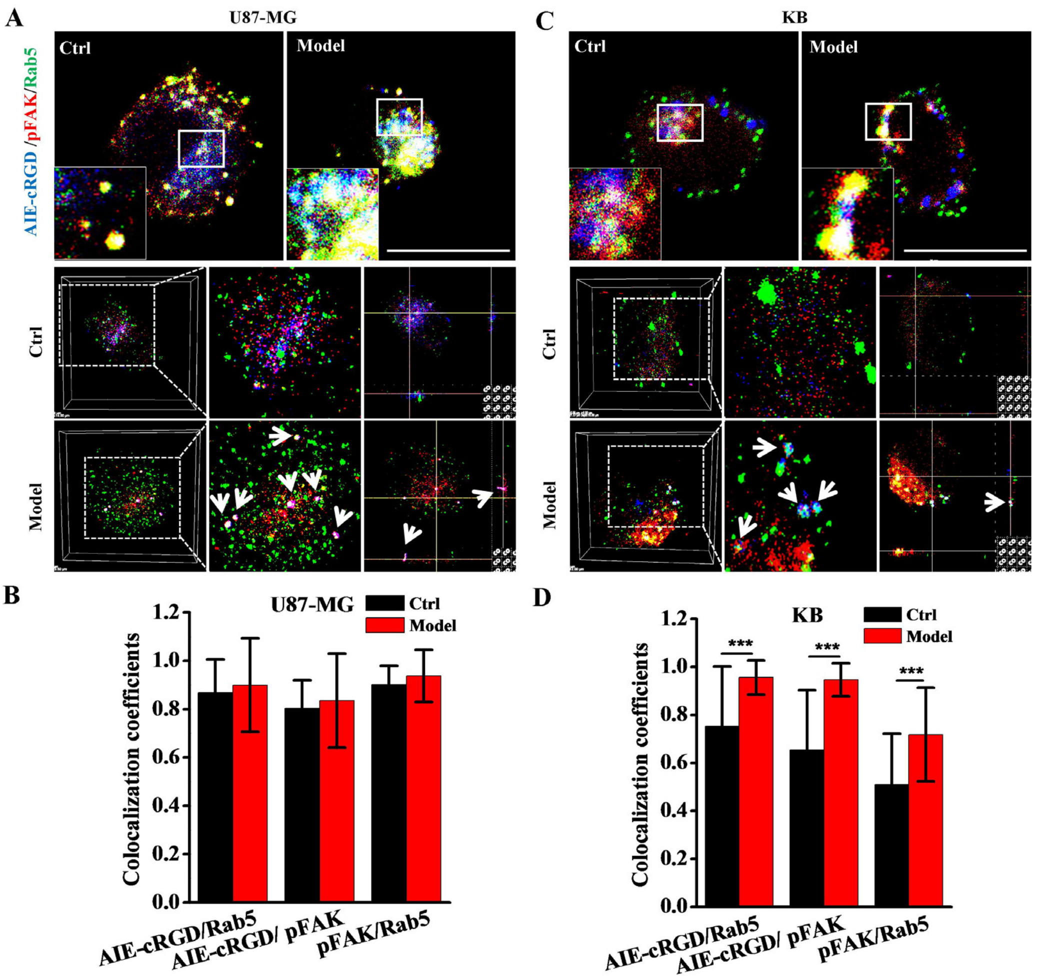

Figure S3. (A) Confocal images of U87-MG stained with AIE-cRGD (blue), fluorescence antibody of anti-pFAK (red), and anti-Rab5 (green). (B) Colocalization rate of U87-MG cells among AIE-cRGD, pFAK, and Rab5. (C) Confocal images of KB cells stained with AIE-cRGD, fluorescence antibody of anti-pFAK, and anti-Rab5. (D) Colocalization rate of KB cells among AIE-cRGD, pFAK, and Rab5. *** $p < 0.001$ represents significant differences compared with the control group. Bar = 20 μm.

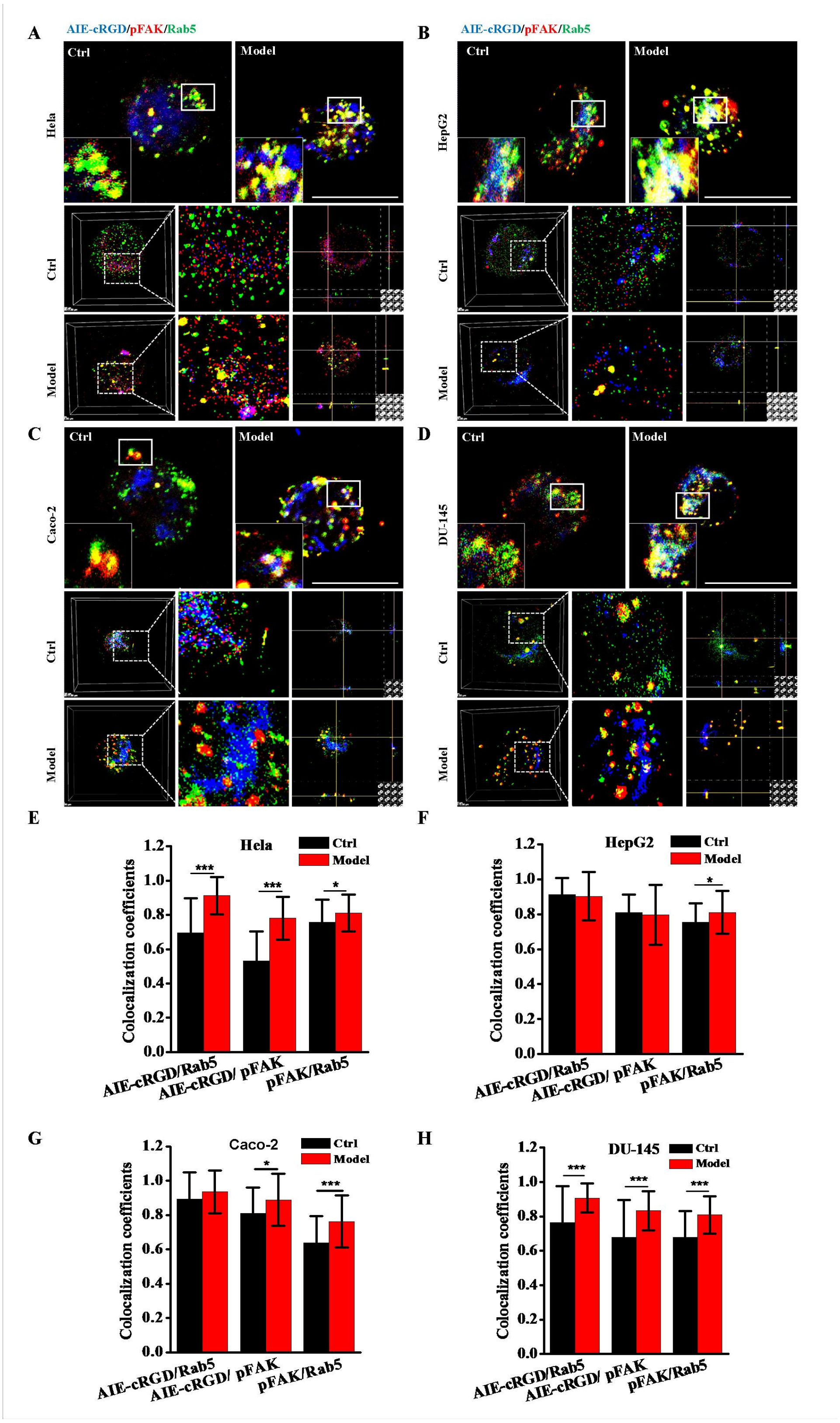

Figure S4. Confocal images of (A) Hela cells, (B) HepG-2 cells, (C) Caco-2 cells, (D) DU-145 cells stained with AIE-cRGD (blue), fluorescence antibody of anti-pFAK (red), and anti-Rab5 (green). Colocalization rate of (E) Hela cells, (F) HepG-2 cells, (G) Caco-2 cells, (H) DU-145 cells among AIE-cRGD, pFAK, and Rab5. *** $p < 0.001$ represents significant differences compared with the control group. Bar = 20 μm.

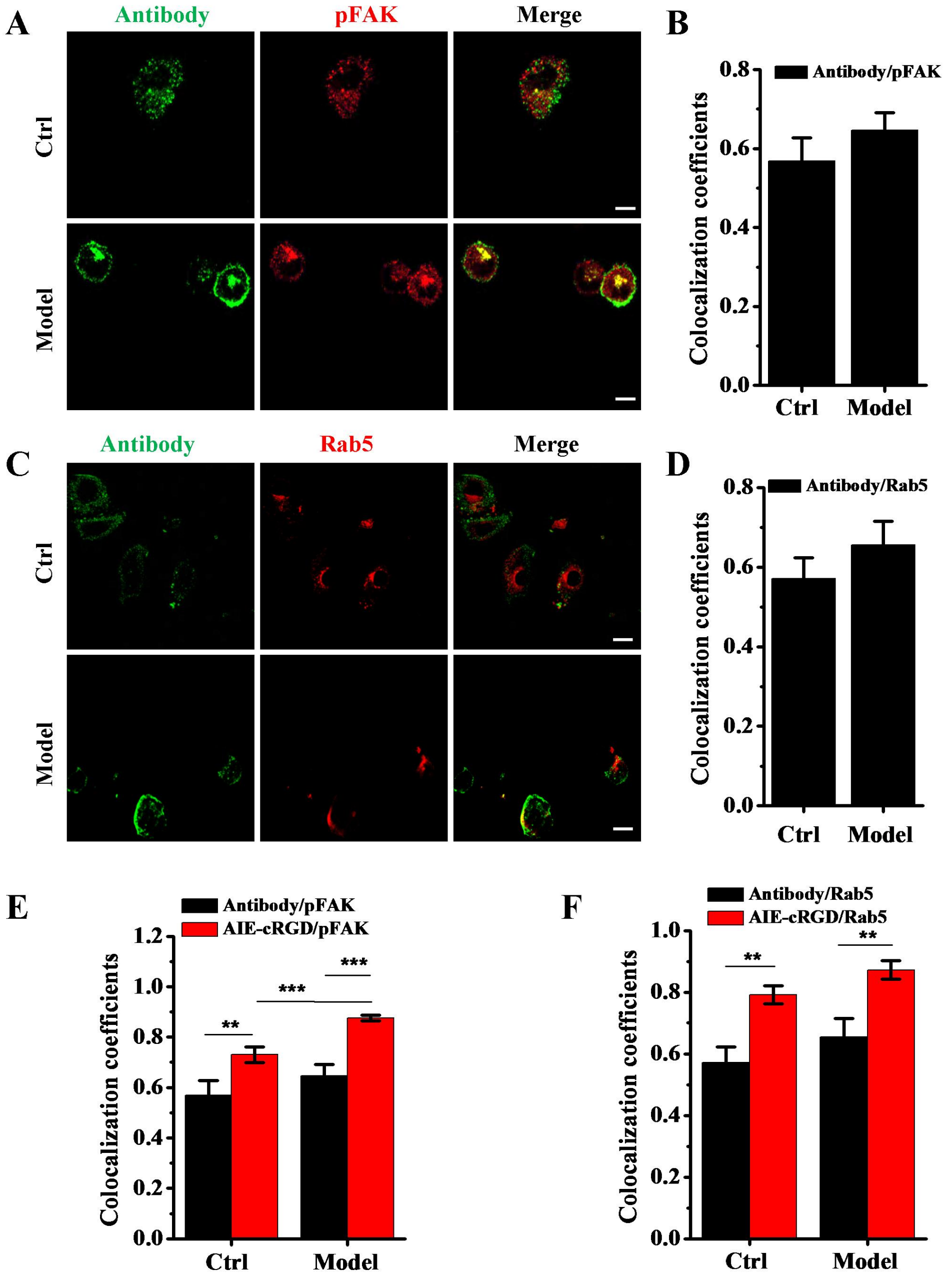

Figure S5. (A) Confocal images and (B) Colocalization statistic of MDA-MB-231 cells stained with anti-integrin antibodies and anti-pFAK antibodies. (C) Confocal images and (D) Colocalization statistic of fluorescence intensity of anti-integrin antibodies and anti-RAB5 antibodies. (E-F) Colocalization analysis in comparison AIE-cRGD with anti-integrin antibody. *** $p < 0.001$, ** $p < 0.01$ represents significant differences compared with the control group. Bar = 20 μm.
